# An Implantable Micro-Caged Device for Direct Local Delivery of Agents

**DOI:** 10.1038/s41598-017-17912-y

**Published:** 2017-12-15

**Authors:** Alexander I. Son, Justin D. Opfermann, Caroline McCue, Julie Ziobro, John H. Abrahams, Katherine Jones, Paul D. Morton, Seiji Ishii, Chima Oluigbo, Axel Krieger, Judy S. Liu, Kazue Hashimoto-Torii, Masaaki Torii

**Affiliations:** 1 Center for Neuroscience Research, Children’s Research Institute, Children’s National Medical Center, Washington, DC 20010 USA; 2 The Sheikh Zayed Institute for Pediatric Surgical Innovation, Children’s National Medical Center, Washington, DC 20010 USA; 3 Terrapin Works, School of Engineering, University of Maryland, College Park, MD 20740 USA; 4 Department of Neurology, Children’s National Medical Center, Washington, DC 20010 USA; 5 Nanocenter FabLab, University of Maryland, College Park, MD 20742 USA; 6 Department of Biomedical Sciences and Pathobiology, Virginia Tech, Blacksburg, VA 24061 USA; 7 Department of Neurosurgery, Children’s National Medical Center, Washington, DC 20010 USA; 8 Department of Mechanical Engineering, A. James Clark School of Engineering, University of Maryland, College Mark, MD 20742 USA; 9 Department of Neurology, Warren Alpert Medical School, Brown University, Providence, RI 02912 USA; 10 Department of Pediatrics, Pharmacology and Physiology, School of Medicine and Health Sciences, George Washington University, Washington, DC 20052 USA; 11 Department of Neurobiology and Kavli Institute for Neuroscience, School of Medicine, Yale University, New Haven, CT 06510 USA

## Abstract

Local and controlled delivery of therapeutic agents directly into focally afflicted tissues is the ideal for the treatment of diseases that require direct interventions. However, current options are obtrusive, difficult to implement, and limited in their scope of utilization; the optimal solution requires a method that may be optimized for available therapies and is designed for exact delivery. To address these needs, we propose the Biocage, a customizable implantable local drug delivery platform. The device is a needle-sized porous container capable of encasing therapeutic molecules and matrices of interest to be eluted into the region of interest over time. The Biocage was fabricated using the Nanoscribe Photonic Professional GT 3D laser lithography system, a two-photon polymerization (2PP) 3D printer capable of micron-level precision on a millimeter scale. We demonstrate the build consistency and features of the fabricated device; its ability to release molecules; and a method for its accurate, stable delivery in mouse brain tissue. The Biocage provides a powerful tool for customizable and precise delivery of therapeutic agents into target tissues.

## Introduction

One of the greatest challenges in modern medicine is the manufacturing of therapies that maximize the precision and personalization for individual patients. The needs for such technologies in medicine are wide-ranging; many, if not most, medical conditions involve the dysfunction of specific organs or the occurrence of diseased regions within otherwise healthy tissues^1^. These include but are not restricted to needs in direct applications for cancer therapy^1–4^; interventions in ocular disorders^5–7^; and treatment of neurological disorders such as Parkinson’s disease^8–10^, Alzheimer’s disease^11–15^, and epilepsy^16,17^, among others^18^. Despite the focal nature of these disease processes, current therapies are delivered systemically, exposing the rest of the body to unwanted side effects and minimizing potential efficacy^19,20^. As such, designing a versatile therapeutic delivery platform that can be precisely tailored for each patient is paramount.

However, the delivery of therapeutic agents into their respective targets presents several challenges. Systemic pharmacological delivery has several limitations as this method non-specifically affects areas and tissues^4,7,21–24^ and must pass through various restrictive blood-tissue barriers^4,6,24–31^. More straightforward solutions have been through the use of implantable devices^3,9,11,13–16,32^ and gels^33–36^ which are applied directly into afflicted regions, maximizing concentrations of local drug delivery while minimizing potential side-effects experienced by systemic delivery options^33–38^. Yet, current devices have several shortcomings in regards to implementations in complex organ systems as they are typically obtrusive and invasive in size, lacking in the fine control necessary for precise delivery, are not customizable for individual patients, and often have added complexities in releasing therapeutic materials^4,14,24,32,39,40^. Therefore, the need for devices that can provide pinpoint precision of therapeutic delivery, maximizes efficacy, reduces side-effects and damage, and accounts for specific patient needs is vital.

One promising technology with immense potential to address these needs is 3D printing and fabrication, whereby delivery methods may be custom-designed with a wide range of materials^41–43^. To date, several applications have utilized 3D printing technologies for biological contexts, particularly in the fabrications of tissues^42,44–51^. More recent approaches have begun to explore the incorporation of therapeutic molecules into biodegradable patches that can be applied to the area of interest^43,46,52–55^. However, the integration of pharmacological agents within these materials is a challenge, as the printing process may affect the stability of the drugs and its ability to elute in surrounding tissues^25,41,54^.

Given these current shortcomings, there exists a need for a customizable drug delivery device that offers both a precise means of delivery along with a flexibility of design. Here, we propose a delivery platform we call the Biocage: a needle-sized perforated container printed at micron-level resolution to be used for the precise and local delivery of therapeutic agents. The device is printed using the Nanoscribe Photonic Professional GT 3D laser lithography system at high accuracy and resolution, can be filled with desired agents, and is robust enough to be implanted directly into target tissues. We demonstrate the consistency and resolution of the printed Biocages; its ability to release materials through its pores; its direct and local delivery into brain tissue; its stability in placement after delivery into tissues; and its effects after *in vivo* implantation. This strategy has the potential for focal and minimally invasive delivery of desired therapeutic agents into tissues of interest, while also offering immense flexibility in designing and filling the device dependent on the situational need.

## Results

### The design of the Biocage: a small device for direct and controlled therapeutic delivery

In identifying a delivery method that provides maximal versatility, we wanted to design a device that (1) is capable of holding therapeutic agents in a fixed container; (2) can release agents over time based on the design of the tool, materials used in its production, and contents of the device; (3) is able to be tailored according to different situational needs; and (4) is local in its direct effect.

**Figure 1:**
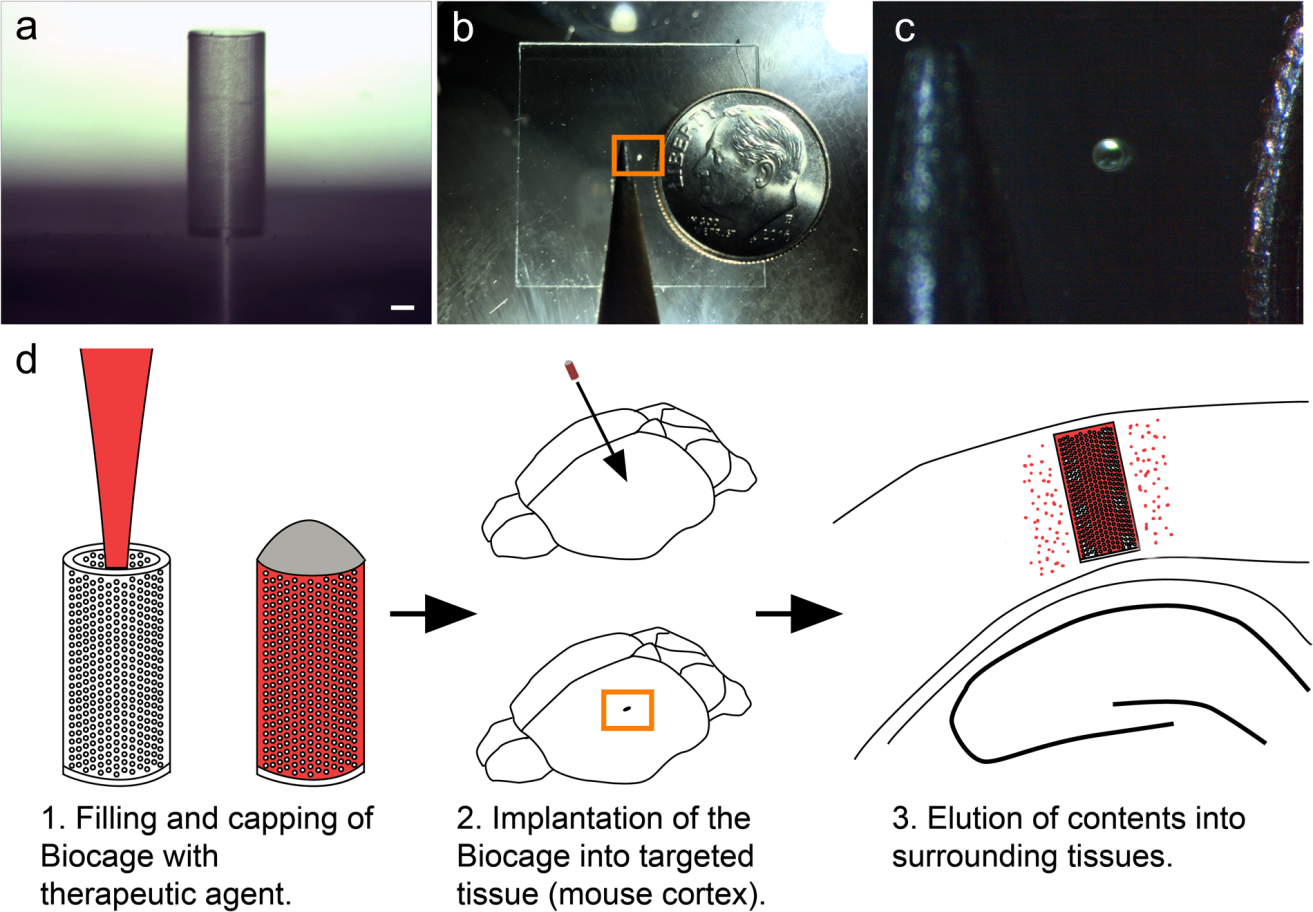
The Biocage and its relative size. (**a**) Light microscopy image of the Biocage device showing its relative proportions and porous exterior. Scale bar = 100 µm. (**b** and **c**) Relative size of the Biocage. Biocage from the top-down viewpoint is displayed in relation to a pencil tip (to the left) and dime (to the right) to reflect its miniature size. (**c**) is a higher magnification of the boxed area in (**b**). (**d**) Schematic of the workflow for implantation of the Biocage. Biocages are first filled with the desired therapeutic molecules and capped. The device is then inserted into the targeted tissue of interest (in our example, a mouse cortex). The contents are allowed to elute into the local surrounding areas of the tissue.

Based on these criteria, we developed a device we call the Biocage (Fig. 1): a hollow cylinder with perforated holes on the exterior designed for the delivery of therapeutic agents for precise and controlled release (Fig. 1a). The device is small enough to fit inside a 22-gauge needle (Fig. 1b and c) to allow for direct delivery. To use, the device is filled with the therapeutic agents of interest, capped, and implanted directly into a target tissue (e.g., the mouse cerebral cortex), whereupon the contents are eluted to the surrounding target area (Fig. 1d).

**Figure 2:**
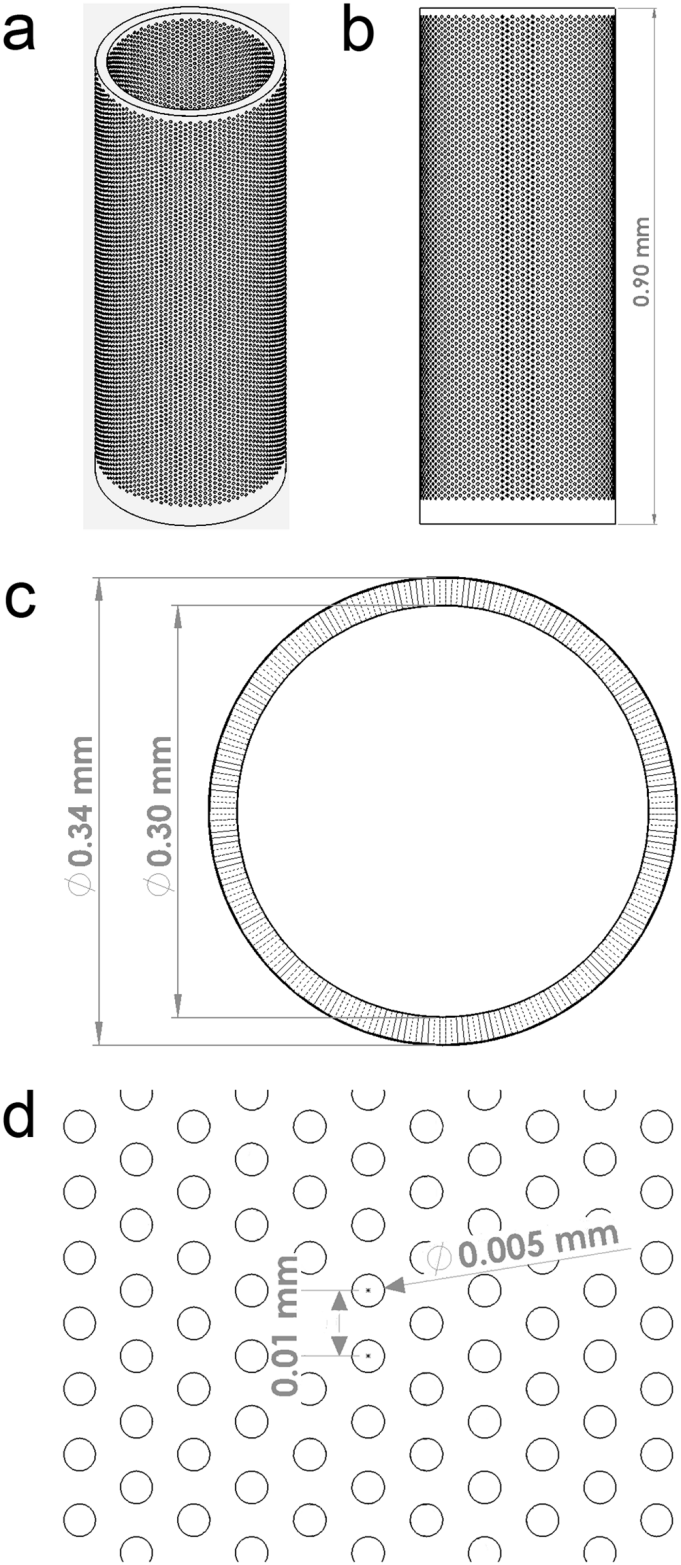
CAD design for the Biocage. (**a**) Oblique angle view of the Biocage. Its design is a 300 µm empty hollow inner diameter to be filled, a 20 µm outer wall, a 40 µm solid base, and a 900 µm total height. The outer wall is perforated with holes, each with a diameter of 5 µm. (**b**) Side profile of the Biocage. (**c**) Top profile of the Biocage showing the holes perforating through the outer wall. (**d**) Higher magnification of pores of the Biocage showing dimensions.

Our design consists of a 300 µm hollow inner diameter, a 20 µm outer wall, a 40 µm solid base, and a 900 µm total height (Fig. 2). The inner diameter was made as small as possible to allow for minimal invasiveness while still being feasibly able to be filled, while the outer wall thickness was made as thin as possible while still retaining the structural integrity of the device. Here, the height was determined to be similar to the thickness of the mouse cerebral cortex^53^ (Fig. 2a and b). We utilized a cylindrical shape, both to be able to be delivered using a standard syringe and needle, as well as to mimic the alignment of cortical columns^56,57^ (Fig. 2c). The outer wall is perforated with holes with a diameter of 5 µm to allow for the release of materials from the device into the surrounding tissue while preventing cells from migrating to the inside of the device (Fig. 2d).

### Fabrication of the Biocage

**Figure 3:**
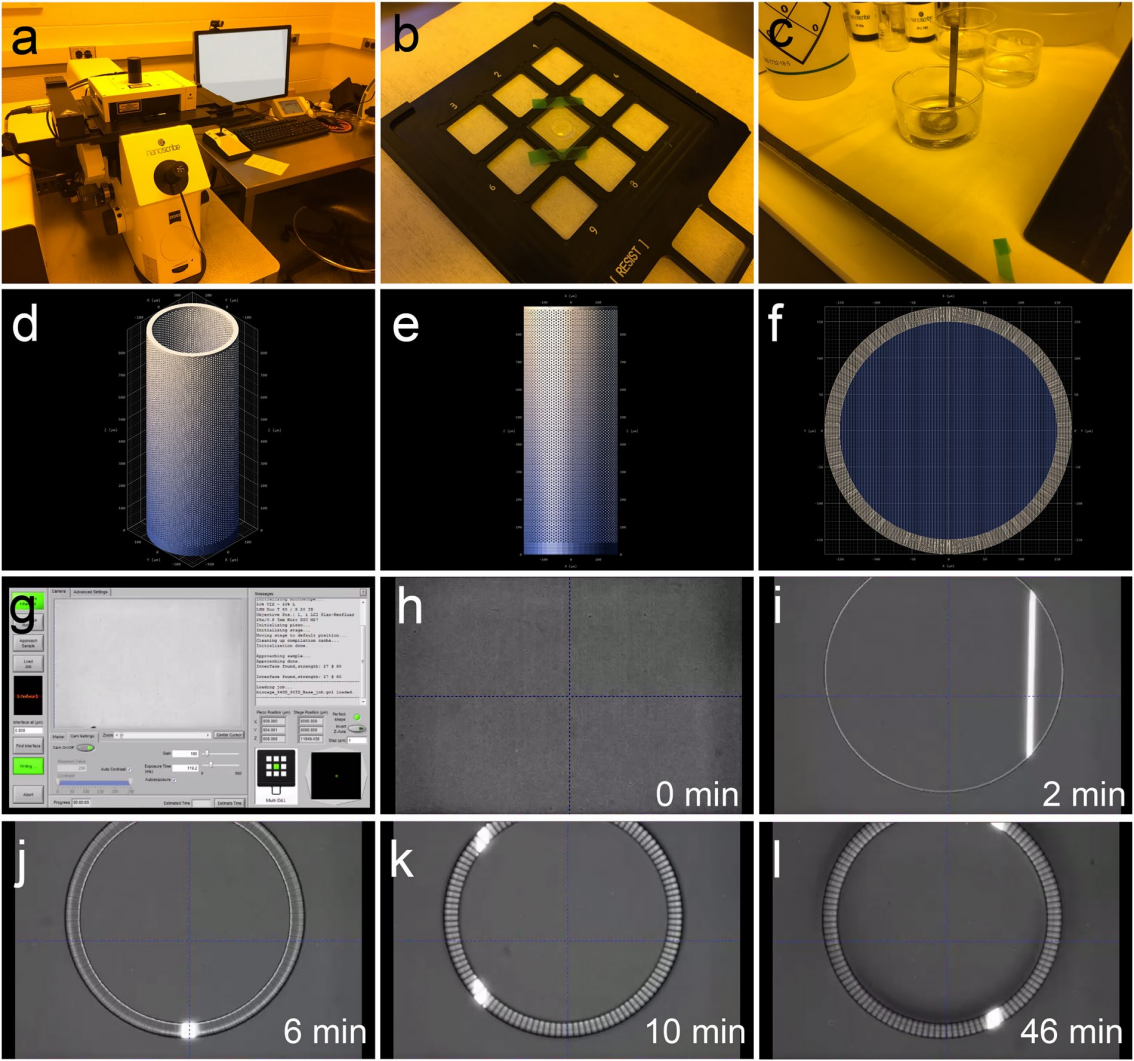
Production of the Biocage using the Nanoscribe lithography system. (**a**–**c**) Printing and development of structures using the Nanoscribe system. Biocages were printed using the Nanoscribe system (**a**). Photoresist was drop-coated onto the substrate for printing (**b**), and was afterwards developed in PGMEA (**c**). (**d**–**f**) DeScribe renderings of the Biocage prior to printing on the Nanoscribe. (**g**) Initial setup screen for Nanoscribe printing using the Nanowrite software. (**h–l**) Time lapse imaging of the Biocage being printed at various time points from the start of printing (**h**) to final completion (**l**). The base of the Biocage is observed at 2 minutes into printing (**i**). The inner wall is then constructed at 6 minutes into printing (**j**). By 10 minutes of printing, the pores of the Biocage begin to be observed (**k**). The total time of making one Biocage is approximately 46 minutes (**l**).

To fabricate the device, we decided on a 3D printing approach that provided adaptability with regards to the materials utilized for printing and the designs that can be made (Fig. 3). We utilized the Nanoscribe Photonic Professional GT 3D laser lithography system^58^, a maskless two-photon polymerization (2PP) 3D printer (Fig. 3a–c). The Biocage was printed directly on a glass substrate with either the photoresist IP-Dip or IP-S (Fig. 3b), after which the cover slip was processed in propylene glycol monomethyl ether acetate (PGMEA) to remove residual materials and subsequently dried (Fig. 3c). This system is especially powerful in its ability to resolve configurations of a wide range, particularly its ability to print structures several hundred microns tall at micron-level resolution^58^ (Fig. 3d–f). In addition, IP-Dip^59,60^ and IP-S^61^ have both been previously utilized for *in vitro* applications, and the mechanical properties of IP-Dip has been previously described^62^. The device was constructed from the bottom-up, with the base of the tube printed first, followed by the walls with the perforated holes (Fig. 3g–l). For the final build, in which we utilized the photoresist IP-S with a 25x objective, the total fabrication time was 46 min (Fig. 3l).

### Build and Consistency of the Biocage

**Figure 4:**
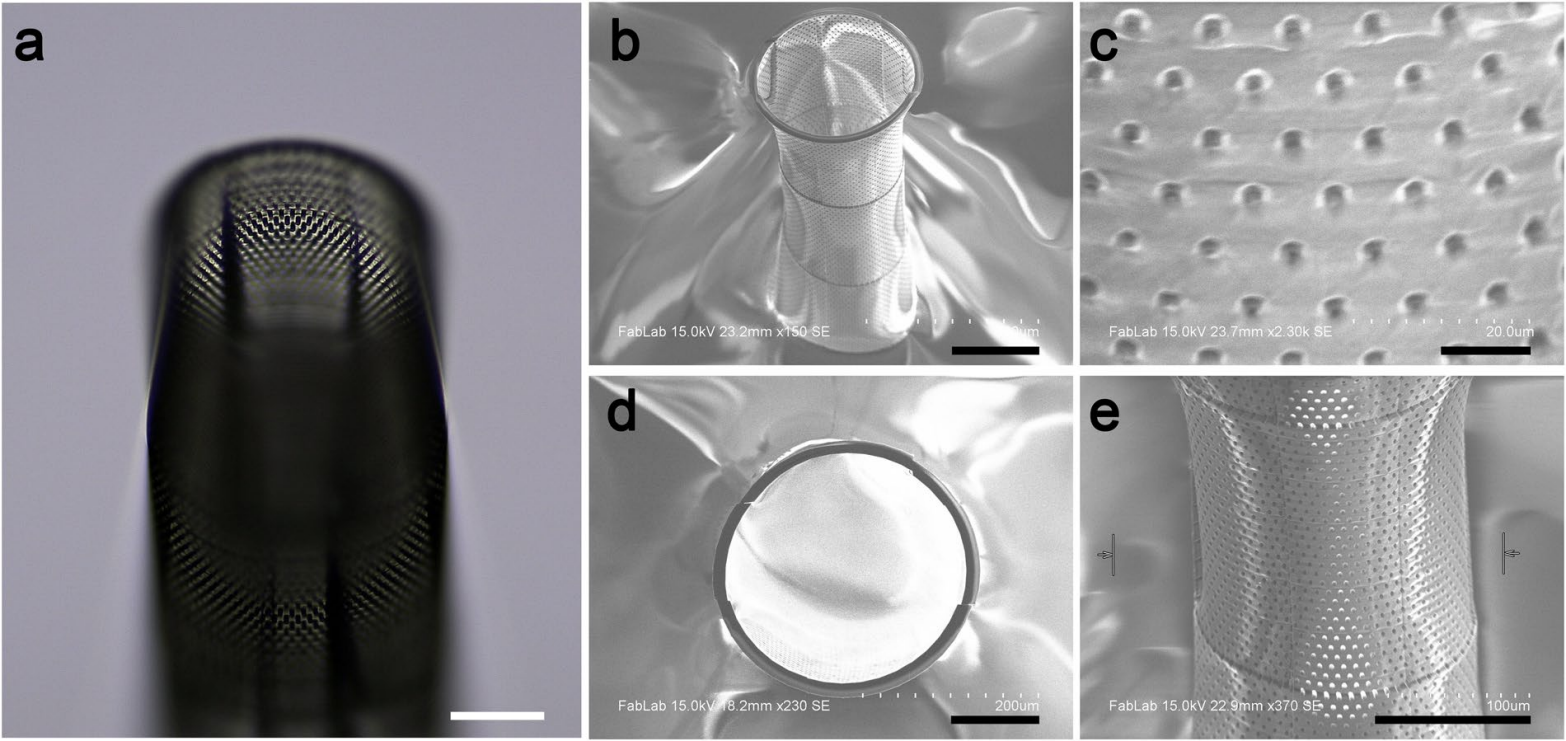
Prototype of the Biocage. (**a**) Oblique light microscope image of the Biocage prototype made with IP-Dip using a 63x objective. The light scattering makes it possible to see the finer features of the device. (**b**–**e**) SEM images of the Biocage prototype. Many of the features, including the hollow tube and holes (**b** and **c**) are present. However, certain aberrations and stitching in printing, including on the rim (**d**) and wall (**e**), were present. Scale bars for **a**,**b**,**d**, and **e** = 100 µm. Scale bar for **c = **10 µm.

Our initial prototypes utilized the photoresist IP-Dip with a 63x objective for printing, which provided a higher resolution print of the device at the cost of efficiency of printing (Fig. 4). Imaging via light microscopy (Fig. 4a) and scanning electron microscopy (SEM) (Fig. 4b–e) showed several of the major features to be present, including the 5 µm holes (Fig. 4c **)**. However, some aberrations were observed including imperfections along the sides and rim of the Biocage and a slight warping of the walls (Fig. 4d and e).

**Figure 5:**
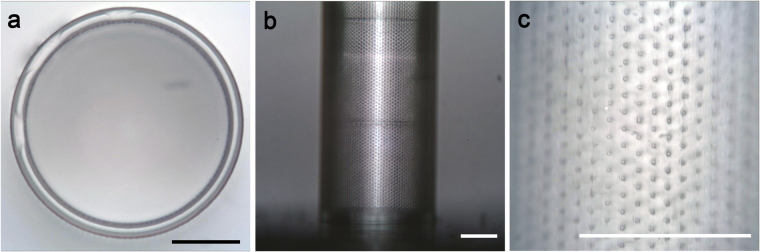
Light microscopy images of final Biocage device. (**a**) The top view shows precision of the wall structure of the device. (**b**) The side profile showing the width and base of the structure. (**c**) Further magnification of the walls shows the fine pore structures of the device. Scale bars = 100 µm.

To eliminate these variations, we tested another condition which was better suited for printing of larger structures, utilizing the photoresist IP-S with a 25x objective (Fig. 5). As the sensitivity of the IP-S material did not allow SEM imaging, we utilized optical microscopy for imaging and measuring the device. With this printing method, Biocages displayed no obvious structural imperfections (Fig. 5a and b) while still retaining the finer features (Fig. 5c). Importantly, these devices showed high levels of consistency in regards to structural integrity and features between builds when analyzing for design error (Table 1). Several of the fine features of the device including pore diameter (Actual: 4.827 ± 0.3938 µm; Design: 5.000 µm; n = 3 devices, 10 holes each device), vertical spacing of pores (Actual: 10.64 ± 0.3938 µm; Design: 10.00 µm; n = 3 devices) and wall thickness (Actual 21.41 ± 1.692 µm; Design: 20.00 µm; n = 5 devices) were consistent to the original design. Macro-dimensions such as the inner diameter (Actual: 307.6 ± 5.644 µm; Design: 300.00 µm; n = 5 devices), outer diameter (Actual: 350.4 ± 6.271 µm; Design: 340.0 µm; n = 5 devices), and height of the devices (Actual: 964.0 ± 9.167 µm; Design: 900 µm; n = 3 devices) showed consistency between devices, but were slightly larger than the original design.

**Table 1:** Biocage physical dimensions and design error in comparison to the original design.

	Actual (µm)	Designed Value (µm)	Design Error (µm)
**Pore Diameter**	4.827 ± 0.3938	5.000	0.173 ± 0.3938
**Vertical Spacing**	10.64 ± 1.969	10.00	0.64 ± 1.969
**Wall Thickness**	21.41 ± 1.692	20.00	1.41 ± 1.692
**Diameter (Inner)**	307.6 ± 5.644	300.0	7.6 ± 5.644
**Diameter (Outer)**	350.4 ± 6.271	340.0	10.4 ± 6.271
**Height**	964.0 ± 9.167	900.0	64.0 ± 9.167

### The release of small molecules through the Biocage

**Figure 6:**
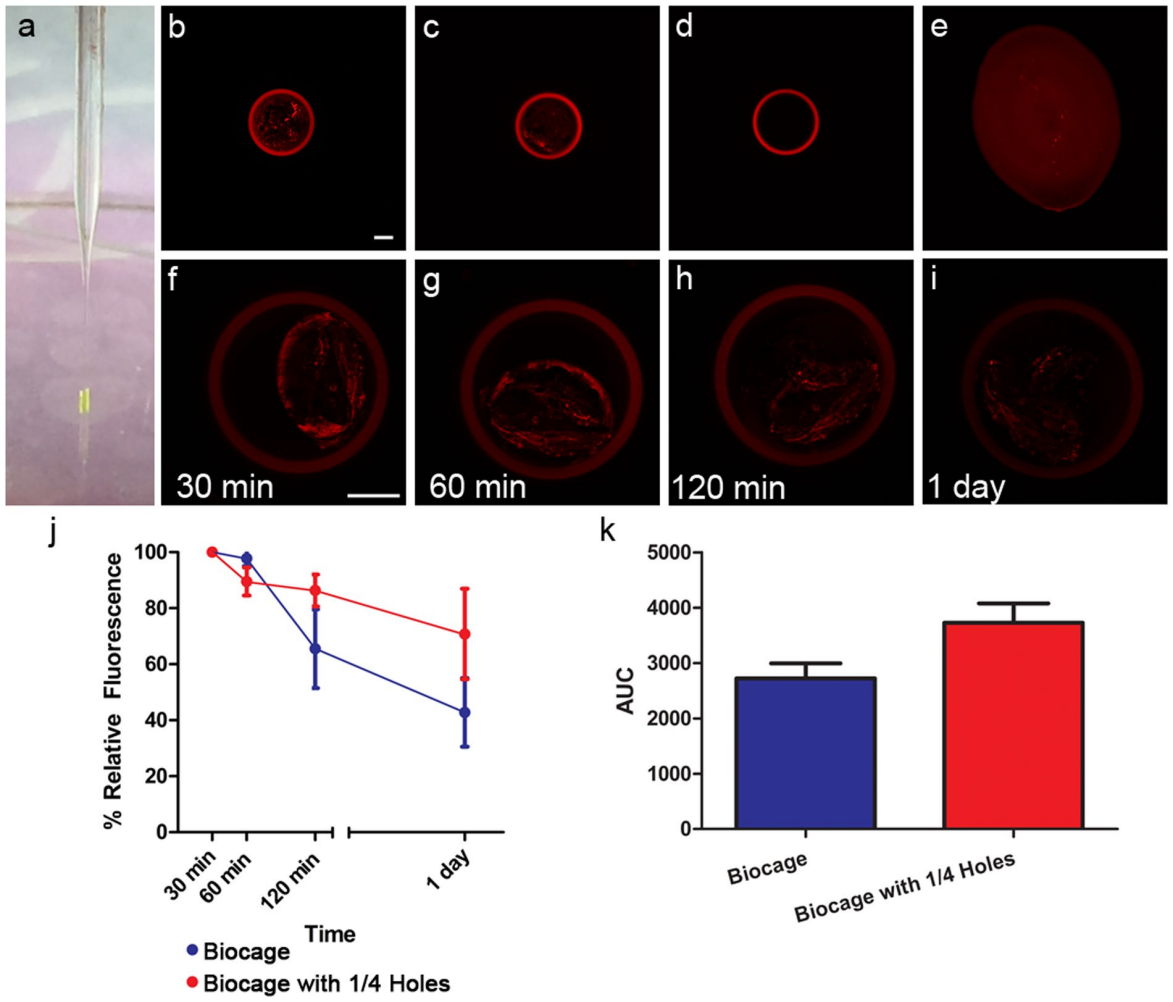
Release of materials through the Biocage device. (**a**) Loading of Biocage device using stereotaxic device and a filled glass pipette. Biocages were filled with fluorescent microspheres at a 1:1000 concentration within a 0.35% agarose solution and allowed to solidify, and sealed with cyanoacrylate adhesive. (**b**–**e**) Biocages are loaded with fluorescent beads in 0.35% agarose. Biocages (**b**) and Biocages with 1/4 of the holes (**c**) show controlled loading of agarose with fluorescent beads. The Biocage alone without the beads (**d**) displays no internal fluorescence. Plating of agarose with fluorescent beads alone (**e**) shows an uncontrolled and variable shape. (**f–i**) Release of fluorescent microspheres from the Biocage. Devices attached to silica glass without bases are filled with fluorescent microspheres in agarose, and put into PBS to demonstrate small molecule release. Beads are imaged using an inverted confocal microscope at equal laser power and exposure times. A reduction of fluorescence is observed (compare **f**,**g**,**h**, and **i**), indicating release of the microspheres. Biocages are autofluorescent, but begin lose this fluorescence when exposed to PBS over extended periods. Scale bar = 100 µm. (**j**) Quantification of relative fluorescent values demonstrating release in both the Biocage (blue) and a Biocage with 1/4 of the number of pores (red). Relative fluorescent values are shown in relation to the initial measure at 30 min in PBS. A trend for release rates are observed in relation to the relative number of pores in the device, with fewer pores resulting in an overall slower rate of release; however, this result was not statistically significant. Error bars in SEM. n = 4 per time point, two-way ANOVA, F_1,23_ = 2.51, p = 0.2396. (**k**) Area under curve (AUC, arbitrary units) of relative fluorescent values versus time. Error bars in SEM. Student’s t-test, p = 0.1951.

We next determined the ability for the Biocage to contain and release material through its pores (Fig. 6). To better measure the rate of delivery, Biocages were filled directly with warmed agarose (an inert polysaccharide hydrogel^63^) mixed with stable 40 nm fluorescent microspheres via a pulled glass pipette and stereotaxic apparatus, after which the contents were dried and the tubes capped with cyanoacrylate adhesive (Fig. 6a). Hydrogels, including agarose, have been extensively established as a means to deliver therapeutic molecules^64–71^. We utilized 0.35% agarose as it provided ample time to fill the warmed liquid into the Biocage and still allowed for hard gelling when cooled. Biocages provided consistent and fixed containment of the agarose mixture in comparison with just the agarose alone (Fig. 6b–e).

To determine whether Biocages were capable of releasing small molecules through its pores, we measured the relative amount of fluorescence within the agarose gel in the Biocage devices soaked in phosphate buffered saline (PBS) over a period of time (Fig. 6f–i). To observe the fluorescence of these molds directly via confocal microscopy, devices in this experiment were printed without the 40 µm base and were directly attached to silica glass to allow for imaging. Given the small volume injected into the Biocage, we allowed the molds to be hydrated in PBS for 30 minutes prior imaging and quantification. The agarose within these devices showed a consistent reduction in fluorescence intensity through a 1 day period relative to their original intensity measured after 30 min in PBS (Fig. 6f–j), indicating the gradual release of the microspheres through the designed pores. When applying the same experiment to Biocages with approximately 1/4 of the number of pores, a similar reduction of fluorescence intensity over time was observed (Fig. 6j). Although not statistically significant, the reduction with this model displayed a trend of diminished reduction associated with the reduced number of pores (Fig. 6j and k).

### Implantation and Stability of the Biocage

**Figure 7:**
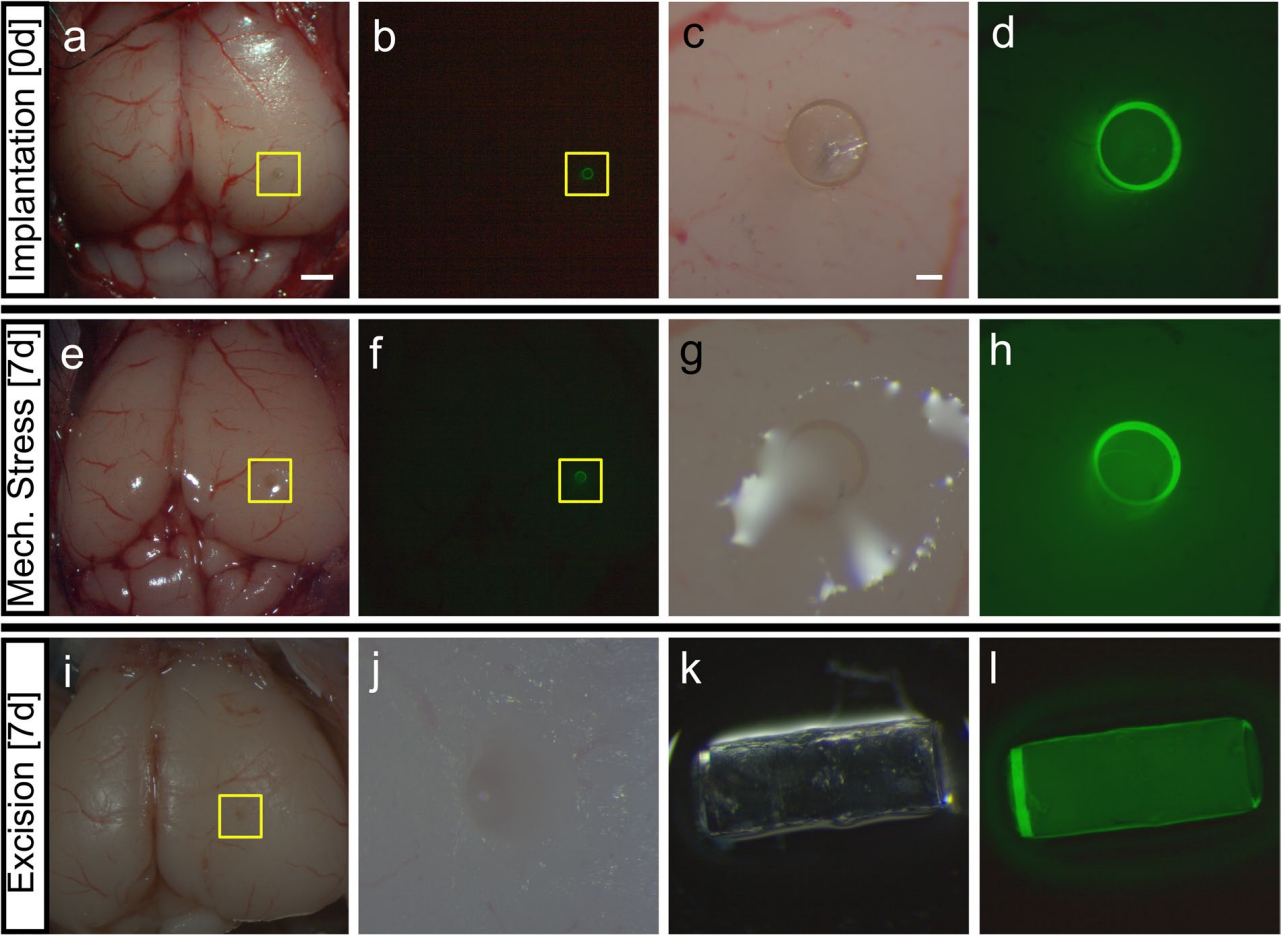
Implantation, mechanical stress, and excision of Biocage in the *ex vivo* mouse brain. (**a**–**d**) Implantation of the Biocage. Light (**a** and **c**) and fluorescent (**b** and **d**) images showing the device in comparison to the rest of the brain. Biocages were filled with 0.35% agarose prior to implantation into *ex vivo* mouse cortex. **c** and **d** are higher magnification views of the areas denoted by yellow boxes in **a** and **b**, respectively. n = 3/3 brains successfully implanted. (**e**–**h**) Light (**e** and **g**) and fluorescent (**f** and **h**) images of implanted Biocages after 7 days of mechanical stress of the same brain shown in (**a**–**d**). Following implantation, brains were fixed with 4% paraformaldehyde overnight to prevent tissue decay, and shook on a circular rocker for a period of 7 days. Biocages were still present within the tissue without movement seen from site of implantation (compare **a** and **b** to **e** and **f**). (**g** and **h**) are higher magnification views of the areas denoted by yellow boxes in **e** and **f**, respectively. n = 3/3 brains. (**i** and **j**) Excision of the Biocage from the tissue. Biocages are successfully excised from the tissue (**i**) without displaying additional damage to the brain (**j**). **j** is a higher magnification of the area denoted by the yellow box in **i**. (**k** and **l**) Light (**k**) and fluorescent (**l**) images of Biocages after mechanical stress and removal. Biocages remained wholly intact after stress and removal. n = 3/3 Biocages successfully excised. Scale bar for a and b, e and f, i = 1 mm. Scale bar for c and d, g and h, k and l = 100 µm.

We next determined the stability of the Biocage and its ability to withstand stressors during implantation and within tissue (Fig. 7). Prior to implantation, Biocages were filled with warmed agarose solution and allowed to solidify, which would both act as a carrier for desired molecules as shown in the small molecule release experiments (see Fig. 6) and provide additional stability for the device to be manipulated. Biocages were then transplanted into *ex vivo* adult mouse brains by first perforating the dura and cortical tissue using the stylet of a solid 22-gauge needle, then inserting the device directly into the perforation. Direct and focal implantation was observed into the mouse brain without difficulty (n = 3/3 successful implanted Biocages); in addition, we were able to track the device through a fluorescence microscopy given the auto-fluorescent properties of the material (Fig. 7a–d).

After successful implantation, we wanted to determine whether the device could withstand other forces within the tissue. In order to mimic mechanical stressors experienced *in vivo*, the implanted *ex vivo* mouse brains were placed in 50 mL conical tubes and shaken on an orbital shaker at 60 rpm continuously for a period of 7 days. The vigorous shaking was done to ensure the safety of the device even when the brain was under such physical duress^72^. After this period, the devices were found within the implanted areas without any observable deficits (Fig. 7e–h, n = 3/3 evaluated implanted brains). This was further evaluated by excising the devices from the tissue and observing both the implantation site and the device itself. Biocages were easily removed, with clear and focal implantation sites observed in the *ex vivo* brains (Fig. 7i and j; n = 3/3 implanted brains). In addition, the Biocages were wholly intact without observable damage, demonstrating the robustness of the device for transplantation (Fig. 7k and l; n = 3/3 excised Biocages).

### *In vivo* Transplantation of the Biocage in the Murine Brain

**Figure 8:**
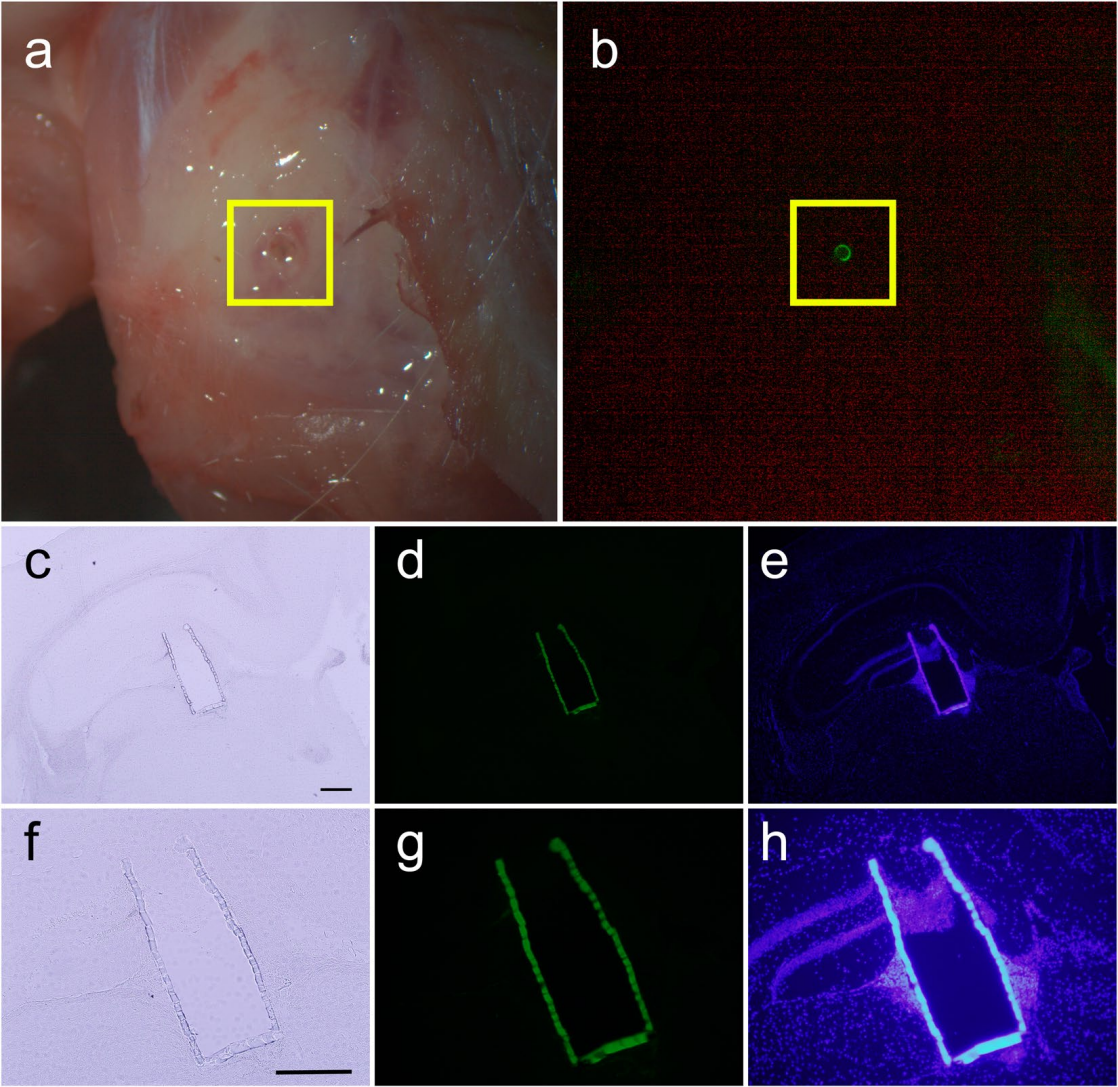
*In vivo* implantation of the Biocage. (**a** and **b**) Uncapped and unfilled Biocages implanted into the cortex after 24 hour implantation *in vivo*. Biocages that are implanted more cortically are directly visible under light (**a**) and fluorescent (**b**) dissecting microscopy. (**c–h**) Coronal brain section showing implanted Biocage within the mouse hippocampus. (**f–g**) are high magnification images of (**c–e**), respectively. Implanted Biocages were cryosectioned directly and is visible under light (**c,f**) and fluorescence (**d and e, g and h**) microscopy. Fluorescence imaging of DAPI staining (**e,h**) shows tissue entering through the uncapped top; however, no cells were observed through the pores along the side wall (**e,h**). Scale bars = 300 µm.

Given the stability of the Biocage observed in the *ex vivo* mouse brain, we next determined the feasibility and stability under *in vivo* conditions (Fig. 8). Adult C57BL/6 mice were anesthetized and had their skulls exposed. A small hole was burred through the skull, after which the dura and tissue was perforated using the stylet of a 22-gauge needle and the uncapped Biocage was placed within the puncture. Animals were allowed to survive for 24 hours prior to sacrifice and analysis. Implanted Biocages were able to be visualized under a dissecting fluorescence scope immediately after perfusion and dissection and observed to be intact (n = 3/3 brains successfully implanted) (Fig. 8a and b) indicating the stability and robust nature of the device *in vivo*. All brains implanted with the device were able to be effectively cryosectioned (Fig. 8c–h). Importantly, while cells are observed to be entering in the uncapped end of the Biocage, they are not observed to be entering through the edges (Fig. 8f–h).

**Figure 9:**
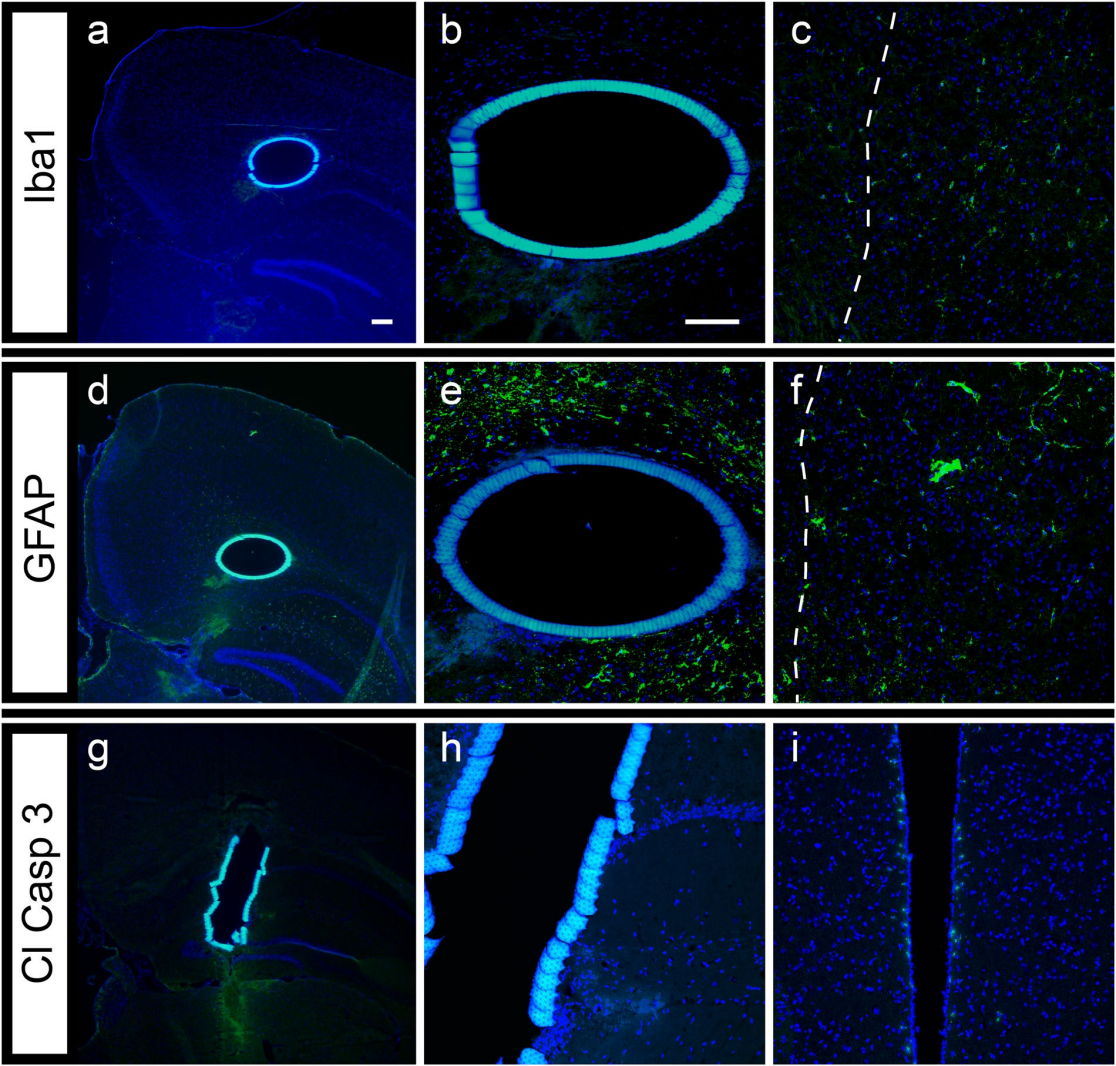
Cellular impact of filled and capped Biocages after 24 hours *in vivo* implantation. (**a–c**) Microglia response observed around the Biocage after 24 hours. Brains with the implanted device were cryosectioned from the top-down view of the device and placed on slides for processing. Staining using the microglia marker Iba1 (**a**) shows few cells around the Biocage (**b**), while several cells are apparent near the needle track (in which the Biocage was implanted; dotted line) (**c**). (**d–f**) Astrocytic response around the Biocage after 24 hours. Staining of cryosections from the top-down view of the device with the astrocyte marker GFAP (**d**) shows a limited astrocyte response immediately around the Biocage (**e**), though similar to the Iba1 labeling, astrocytes were apparent around the needle track region (dotted line) (**f**). (**g–i**) Cell death around the Biocage after 24 hours. Cryosections of the side view of the device shows few cells positively labeled for cleaved Caspase 3 (Cl Casp 3) (**g**) observed around the Biocage (**h**). Staining was observed in other areas of the brain where normal programmed cell death is occurring, including the fourth ventricle (**i**). Scale bars = 100 µm.

We next looked at the cellular effects of the implanted Biocage after 24 hours (Fig. 9). For this experiment, Biocages were loaded with 0.35% agarose and capped to mimic the delivery of agents into the brain. Immunohistochemical examination was done to identify microglia and reactive astrocytes by using antibodies against IbaI (Fig. 9a–c) and GFAP (Fig. 9d–f), respectively. Few to no Iba1 and GFAP positive cells were found around the device, even while the cells expressing these markers were clearly present within the needle track used to penetrating the cortex to allow for the insertion of the Biocage. In addition, we also observed minimal labeling of cells with cleaved caspase-3 (Cl Casp 3), a marker for cell death, around the implantation site, a result similarly observed from other reported injection paradigms^73^ (Fig. 9g and h) while staining was observed in other areas where normal programmed cell death is occurring including the fourth ventricle (Fig. 9i)^74^. Cells were not observed within these filled and sealed devices. These data indicate that implantation of the Biocage after 24 hours results in minimal neural damage and immune response.

### The Delivery of Loaded Agents from the Biocage 24 Hours After *in vivo* Implantation

**Figure 10:**
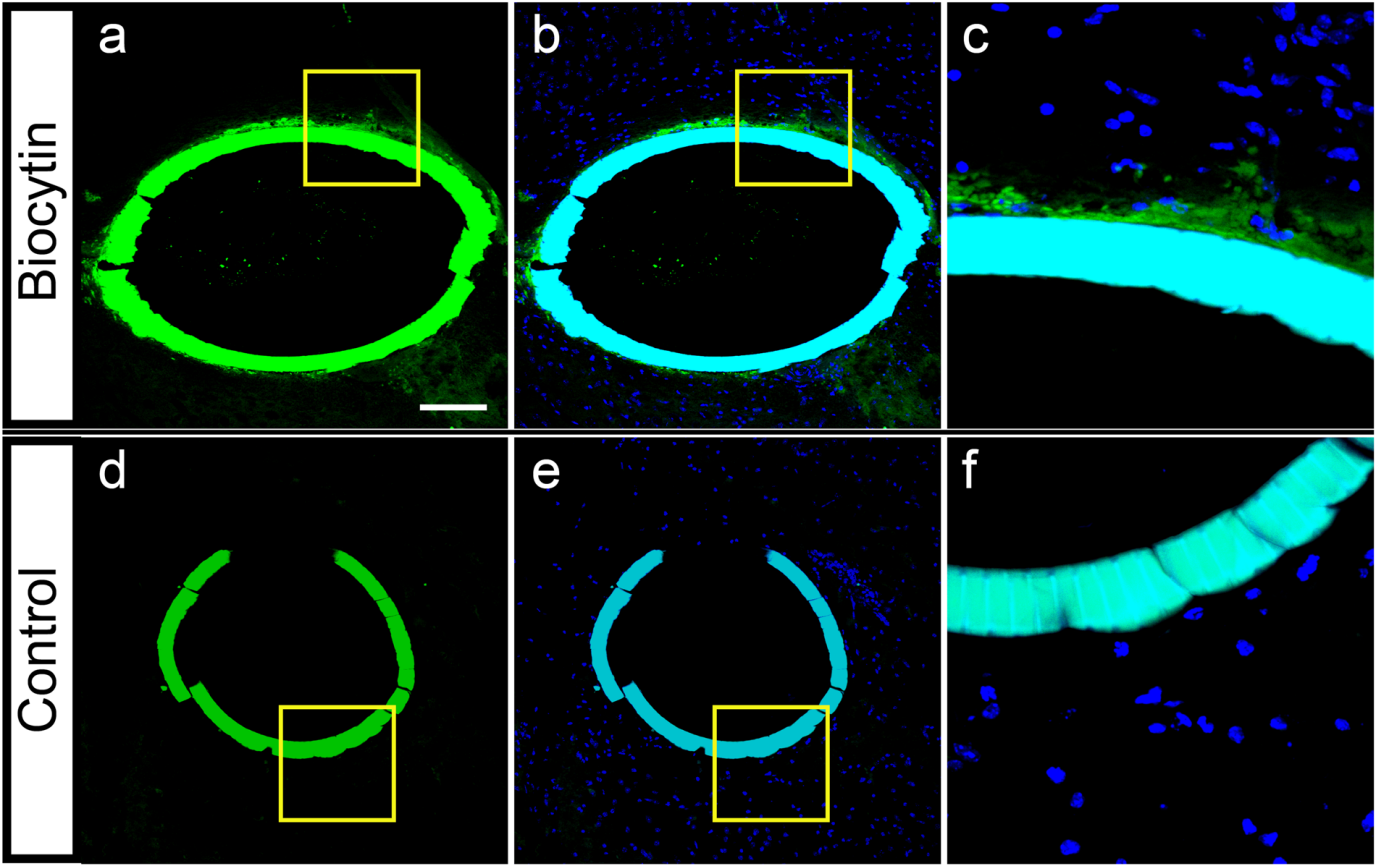
Release of biocytin from the Biocage after 24 hour *in vivo* implantation. (**a–c**) Biocages were filled with biocytin in 0.35% agarose and capped. Biocytin labeling is visible in both the Biocage and areas immediately surrounding the Biocage (**a**, green puncta). Cells are only observed surrounding the enclosed Biocage (**b**, counterstain with DAPI for nuclei). (**c**) Is a higher magnification of the area denoted by the yellow box in (**a** and **b**) showing the biocytin released from the Biocage. (**d–f**) Control Biocage only filled with 0.35% agarose and capped. No puncta is observed within or outside of the Biocage (**d**). Cells are also not observed within the Biocage (**e**). (**f**) is a higher magnification of the area denoted in (**d** and **e**). Scale bar = 100 µm.

We next determined whether it was possible to release materials loaded from the Biocage into surrounding tissues after implantation (Fig. 10). To determine release of small molecules in an *in vivo* environment, the Biocages were loaded with biocytin, a low molecular weight (M.W.: 372.48) conjugate of biotin and lysine^75,76^, in a 0.35% agarose solution, after which they were capped and implanted into cortical brain tissue. Animals then recovered from the surgery, after which the tissue was collected 24 hours after implantation. Biocytin release was clearly observed in the areas surrounding the Biocage (n = 3/3 brains successfully implanted and demonstrating biocytin labeling on the exterior of the Biocage) (Fig. 10a–c), the labeling of which was not observed in controls Biocages that were only loaded with agarose (Fig. 10d–f), demonstrating the ability for our device to release agents within an *in vivo* environment.

## Discussion

Here, we report the fabrication, loading, implantation, and functionality of the Biocage, a porous micro-container designed for focal and precise delivery of therapeutic agents into tissues. The Biocage device is constructed using a 2PP laser lithography system that allows a range of printing of features at high resolution from sub-micron to millimeter scale. We further demonstrate that the device is capable of being filled and can elute substances through its pores and has the robustness of being directly implanted into brain tissue in a precise manner while maintaining its integrity after experiencing external forces for an extended period of time. Finally, we demonstrate the implantation of the Biocage into an *in vivo* brain, its stability and cellular consequences, and its functionality within an *in vivo* environment after 24 hours. Our current data indicates the Biocage to be a biosafe system that results in limited tissue damage and immunological response capable of releasing molecules within the brain. Future goals will be to further define the effects of this device in an *in vivo* context for extended periods of time, optimize the parameters in releasing chemical and molecular agents from the device, and identify the means by which this focal delivery method can alleviate the symptoms of specific disorders.

Our ultimate objective is to develop a versatile, controlled, and precise means of delivering therapeutic agents into diseased or afflicted tissues. The need for exact local delivery devices has been of a particularly strong consideration for addressing diseases which would benefit from precise treatments including in regards to neurological disorders and injuries^8–18^, ocular diseases^5–7^, and cancers^1–4^. Utilizing high resolution 3D printing technology on a micron scale, as demonstrated here, provides a unique opportunity in creating customizable devices that can precisely deliver these therapies with direct and pinpoint accuracy. Precise local delivery of therapeutic agents can potentially maximize the necessary beneficial effects that would otherwise be toxic or unsafe depending on the context of the therapy^37,38^. For instance, utilizing higher concentration doses of therapies that would otherwise be toxic for treatments in small volumes with the Biocage may be possible, such as chemotherapies^77,78^ or nanoparticles^79^, by exposing the target of interest directly with the agent of interest while limiting its effects within other body systems.

The Biocage has immense potential in addressing clinical conditions of complex and sensitive systems. One example for this is in the targeted delivery of restorative therapies to the central nervous system, in which current therapeutic limitations are related to the challenges posed by the complexities of the system and its restricted reserves for regeneration and growth^80,81^. Ideally, treatments would include a means of delivering neuromodulatory agents to moderate brain development and function by altering neurobiological phenomena^82^. However, saturation of the network with these agents by systemic application will not achieve the neural network dynamics required for targeting specific nodes relative to the rest of the system^82^. A precise implant, as described here, would allow for the targeted delivery of these neuromodulatory agents to specific nodes in the central nervous system. This, along with current advances in stereotactic neurosurgical techniques^83^ and intraoperative advanced imaging^84^, has the potential for the highly precise delivery of implants into specific areas within the brain. Focused invasive interventions may help bridge these biological distances especially if specific defective “nodes” in an extensive neural network are targeted.

### The Adaptability of the Biocage Technology

**Figure 11:**
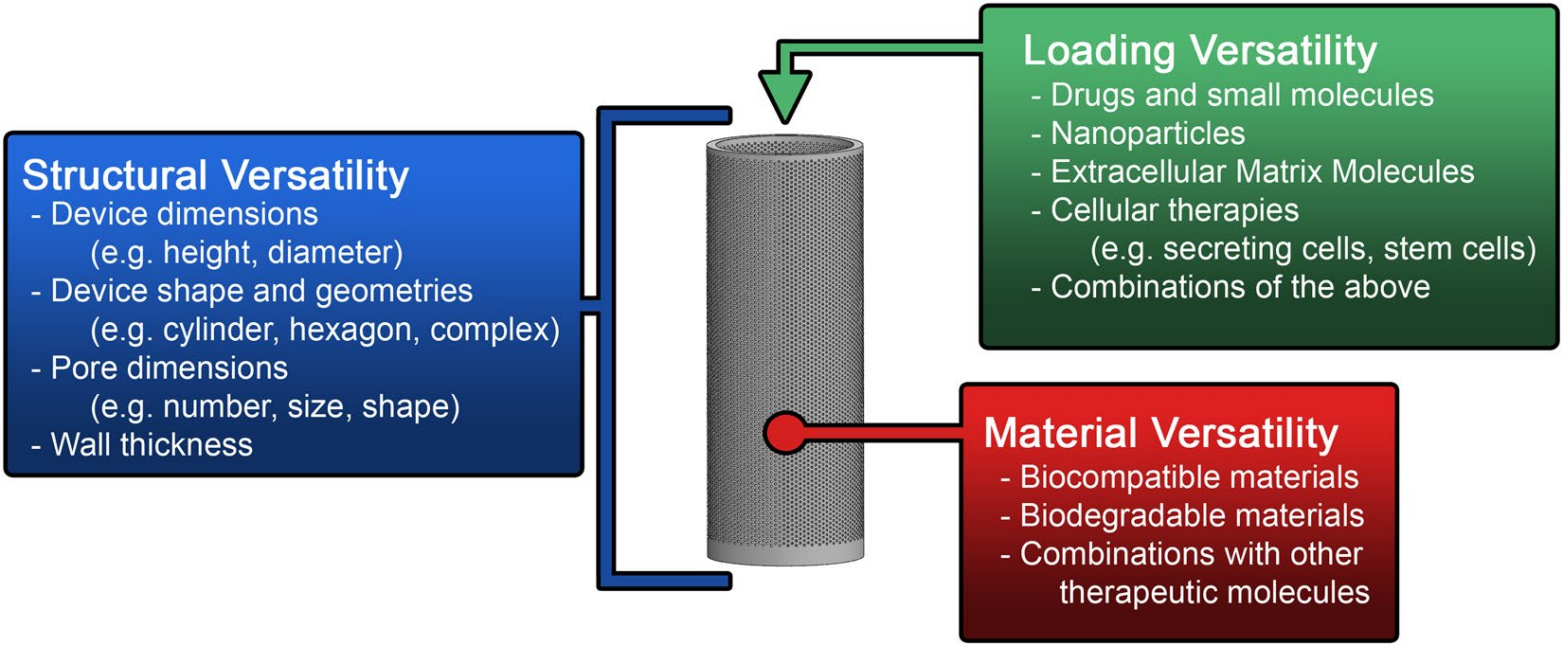
Versatility of the Biocage. Several attributes of the Biocage can be easily modified given its design and production for personalized use. Structural versatility comes in the form of attributes of the design including the length, diameter, and shape of the device. Material versatility is possible through different materials that can be used for 3D printing. Loading versatility allows for several different applications.

Given the varied needs for delivery platforms in several fields, a critical consideration for the development of this device was to identify a tool for delivering therapeutic agents with the most versatility possible while still retaining an overall simplicity that can be utilized in a practical fashion. As such, both our method of fabrication and overall design provides an extensive range of adaptability in several regards (Fig. 11). The 3D printing of our device makes it possible to implement alterations in the dimensions required including shape, size, thickness, and porosity (Structural Versatility). Additionally, the production method provides versatility based on the materials for fabrication, including the possibility of utilizing biodegradable polymers or combining these materials with other agents (Material Versatility). This provides for greater possibilities of utilizing materials combined with therapeutic molecules for additional delivery options. Finally, the ability to fill our device and allow the release of its contents provides a truly unique opportunity of easily combining this local delivery system with multiple other techniques and technologies for controlled drug delivery (Loading Versatility).

This versatility provides not just an advantage in the problems which the Biocage may be able to address, but may also be utilized to circumvent limitations of other technologies. For instance, direct injection systems such as biodegradable microneedles often have limitations in their therapeutic applicability as a result of the harsh fabrication methods that may damage or denature the molecules of interest^85^. The Biocage design circumvents many of these issues by being able to directly load drug within the central chamber for release. In the event that biodegradable materials are utilized in the future, there is a possibility for the Biocage to be able to release therapies both through the loaded chamber and within the dissolvable material itself^85^, providing more therapeutic options within a single device. In this regard, our device can potentially provide an incredibly tightly regulated means of control for therapeutic delivery for multiple applications based on both the properties of the device itself as well as its contents, without solely depending on complex release systems^39,40^.

Furthermore, this device may also provide a means of other therapies that have had faced certain technical limitations, including stem cell and gene therapies^18,86–88^. In these cases, inappropriate cellular activities and micro-environments have often attributed to limited successes. Creating a fixed and conducive local environment within a Biocage may provide a means of making these therapies more realistic for patient use in the future.

As a result of our consideration for the design of the device and its method of production, the device itself may therefore be designed to the requirements of the situation or patient on-demand straight from printer-to-patient. It may be feasible to image the afflicted targeted region or tissue, fabricate a custom-made device based on these dimensions in a short time frame (in our case, less than 1 hour), have its contents exactly customized according to the therapeutic needs, and directly delivered to the region of interest, all within a day.

Given its potential, much research remains in terms of properly utilizing the Biocage for various therapeutic contexts. The fabrication of the Biocage currently is done using IP-Dip^59,60^ and IP-S^61^, both of which have been previously used in *in vitro* contexts. However, the biological safety of these materials has not been formally tested and may not be ideal for implantation for clinical use. Future aspects to enhance this technology will focus on using other biocompatible and biodegradable materials for printing the device.

In addition, other properties of the Biocage, including the modeling of diffusion dynamics of drug dose concentrations and integration other therapeutic technologies for precise delivery, will need to be researched in order to utilize the Biocage for maximal benefit in different clinical contexts as discussed earlier. In this current study, we had not observed a significant difference in the rate of molecule release after the reduction of pores to 1/4 of the number from the original design (see Fig. 6). In this experiment, given the small volume injected into the Biocage, we allowed the agarose containing fluorescent microspheres to be hydrated in PBS for 30 minutes prior to imaging, and the fluorescence intensity of the tube contents at each time point was calculated relative to the intensity at this point of 30-minute hydration. This technically precluded evaluation of the potential burst release of microspheres that may occur during the initial 30 minutes, and thereby may have underestimated the impact of differential pore sizes of the Biocage on the release rate. In any case, altering other characteristics including the carrying medium or hydrogels within the bore of the device, the size of the pores, or even more drastically reducing the number or location of the holes may be able to provide further control for molecule release. Identifying and addressing its limitations, such as seeing its viability with low viscosity drugs and the survivability of cells within the local device environment, will be important for adapting the device for its respective applications.

Together, the Biocage device is a powerful tool for local and precise therapeutic delivery in tissues. The flexibility in its design, materials, and the contents that can be filled provides an important opportunity for the delivery of direct therapies in a customizable, controlled, and exact manner.

## Methods

### Animal Handling

All animals were handled in accordance to protocols approved by the Institutional Animal Care and Use Committee of Children’s National Medical Center (IACUC Protocol #: 00030626). All methods were performed in accordance with the relevant guidelines and regulations.

### Generation of CAD Design

Solidworks 3D CAD software (Dassault Systèmes SolidWorks Corporation, Waltham, MA**)** was used to design and model iterations of the Biocage. Pores were generated by projecting a linear pattern of circles on the inner surface of the Biocage, revolving the linear pattern around the central axis, and performing an extrude cut feature to remove material equivalent to the wall thickness. The model is exported as a binary stereolithography (STL) file for 3D printing. A deviation tolerance of 0.50 µm and angular tolerance of 10 deg was chosen to preserve resolution during fabrication. The Biocage was designed with a total of 10,140 pores on its exterior. For small molecule release experiments, the Biocages with 1/4 holes were designed with a total of 2,550 pores.

### Fabrication of the Biocage

Biocages were printed using the Nanoscribe Photonic Professional GT 3D Laser Lithography system (Nanoscribe GmbH, Eggenstein-Leopoldshafen, Germany). Printing file was prepared using DeScribe software (Nanoscribe GmbH), and printing was done using Nanowrite software (Nanoscribe GmbH). Photoresists used were IP-Dip printed on fused silica glass substrate (for the 63x objective) and IP-S on ITO-coated soda-lime glass (for the 25x objective) (Nanoscribe GmbH). Photoresist was drop-coated on substrates cleaned with 99% acetone, 99% isopropyl alcohol, and ddH2O, and was placed directly in contact with the objective using Dipin Laser Lithography (DiLL) printing mode. Printing was done with a printing speed of 20,000 µm/s at a laser power of 35 mW. After printing, the samples were post-processed in propylene glycol monomethyl ether acetate (PGMEA) for 30 min to remove excess uncured photoresist, washed with 99% isopropyl alcohol, and air-dried.

### Imaging and Sample Processing

Scanning electron microscope imaging (SEM) was done using a Hitachi S-3400N (Hitachi, Tokyo, Japan). Optical imaging was done using a Leitz Wetzlar Ergolux Microscope model 020–488.026 (Leica, Wetzlar, Germany) with TSview7 software (Xintu Photonics, Fujian, China), and measurements for the Biocage were done using ImageJ^89,90^. Pore diameters were measured for both a horizontal and vertical measurement and averaged. Vertical pore spacing was measured by finding the distance between the center of one pore and the center of the vertically adjacent pore. For these measurements, 10 measurements were made for every tube, after which an average measurement was calculated and compared to other tubes.

### Filling of Biocages

Biocages were loaded with the aid of a stereotaxic apparatus (Kopf Instruments, Tujunga, CA) and a pulled 1 × 90 mm glass capillary (Narishige, Tokyo, Japan). The tip of the pipette was broken and attached the stereotaxic apparatus parallel to the barrel of the Biocage. To fill Biocages with agarose solution, 2% agarose in PBS was first melted and diluted to room temperature PBS at a final concentration of 0.35%. This solution was loaded into the pulled glass pipette and stereotaxically inserted into the barrel of the Biocage. Its contents were then released into the chamber of the device via a Picospritzer (Parker, Hollis, NH), after which the mold was allowed to dry and solidify. Biocages were subsequently capped with cyanoacrylate adhesive to seal its contents.

### Small Molecule Release Experiments

To determine the release of molecules from the Biocage, 0.04 µm (40 nm) Fluosphere Fluorescent Microspheres (Thermo Fisher Scientific, Waltham, MA) were mixed with the 0.35% agarose solution at a 1:1000 concentration and loaded into devices without the 40 µm base attached to silica glass. The molds were allowed to solidify and dry, and Biocages were subsequently capped with cyanoacrylate adhesive to seal its contents. Filled devices were then placed in a 60 mm Petri dish and submerged in 5 mL of PBS to allow for small molecule release. Given the small volumes utilized, the agarose was hydrated in PBS for 30 minutes prior to imaging. Fluorescence was captured using an Olympus FV1000 (Olympus, Tokyo, Japan) confocal microscope at the indicated times within PBS, and relative fluorescence intensity was determined using ImageJ software. Percent (%) Relative Fluorescence Intensity was calculated by the relative fluorescence intensity of the tube contents at the indicated time point divided by the relative fluorescence intensity of the tube contents at 30 minutes times 100%.

### Implantation and Mechanical Stress Testing

For *ex vivo* implantation tests, adult wild-type C57BL/6 mice (The Jackson Laboratory, Bar Harbor, ME) were sacrificed, with the top of the skull removed to expose the cerebral cortex. The stylet of a 22-gauge needle was used to puncture a small opening in the cortex. Biocages filled with 0.35% agarose were then placed into the cortical regions. For mechanical stress testing, *ex vivo* mouse brains with implanted Biocages were placed in 50 mL conical tubes. Tissue with the Biocage was first fixed overnight with 4% paraformaldehyde in PBS, and then subsequently washed in PBS 3 times 10 minutes each. The conical tubes were then filled with 40 mL of PBS and placed on an orbital shaker at 60 rpm continuously for 7 days. Brightfield and fluorescence imaging of the Biocage was done using a Zeiss SteREO Discovery V8 microscope (Carl Zeiss, Oberkochen, Germany).

For *in vivo* implantation, Biocages were loaded as described with agarose prior to animal surgery. For biocytin release experiments, Biocages were loaded with 5 mg/mL biocytin (Sigma-Aldrich, St. Louis, MO) in agarose prior to *in vivo* implantation. Adult C57BL/6 mice were anesthetized with ketamine/xylazine (100/10 mg/kg), after which the skull was exposed with a sterile scalpel, and a small section of brain exposed using a surgical drill with a 0.7 mm diameter burr (Fine Science Tools, Foster City, CA). Cortical brain tissue was pierced with the stylet of a 22-gauge needle, after which Biocage was inserted directly into the pierced region. After implantation, animals were allowed to recover for 24 hours after which they were transcardially perfused with 4% paraformaldehyde.

### Histology and Immunohistochemistry

Brains were fixed with 4% paraformaldehyde in phosphate buffered saline (PBS) overnight, rinsed in PBS the following day, and were subsequently placed in 30% sucrose in PBS overnight. The cryopreserved brains were then placed in Optimal Cutting Temperature (OCT) compound (Sakura Finetek, Torrance, CA) and frozen on dry ice, after which they were sectioned into 12 µm coronal slices. The following primary antibodies were used: rabbit polyclonal anti-IbaI (1:500; Wako Laboratory Chemical, Osaka, Japan), anti-GFAP (1:500; Agilent Technologies, Santa Clara, CA), anti-cleaved caspase-3 (1:500; Cell Signaling Technology, Danvers, MA). Immunohistochemistry was performed as described previously^91^. For biocytin detection, sectioned tissue was stained using the ABC-HRP kit (Vector Labs, Burlingame, CA) and TSA Fluorescein (PerkinElmer, Waltham, MA). Sections were nuclear counter-stained with DAPI (Molecular Probes, Eugene, OR).

## References

[1] Mirnezami R, Nicholson J, Darzi A (2012). Preparing for precision medicine. The New England journal of medicine.

[2] Klinghoffer RA (2015). A technology platform to assess multiple cancer agents simultaneously within a patient’s tumor. Science translational medicine.

[3] Jonas O (2015). An implantable microdevice to perform high-throughput *in vivo* drug sensitivity testing in tumors. Science translational medicine.

[4] Huynh GH, Deen DF, Szoka FC (2006). Barriers to carrier mediated drug and gene delivery to brain tumors. Journal of controlled release: official journal of the Controlled Release Society.

[5] Gaudana R, Ananthula HK, Parenky A, Mitra AK (2010). Ocular drug delivery. The AAPS journal.

[6] Davies NM (2000). Biopharmaceutical considerations in to*p*ical ocular drug delivery. Clinical and experimental pharmacology & physiology.

[7] Short BG (2008). Safety evaluation of ocular drug delivery formulations: techniques and practical considerations. Toxicologic pathology.

[8] Kordower JH (1999). Clinicopathological findings following intraventricular glial-derived neurotrophic factor treatment in a patient with Parkinson’s disease. Annals of neurology.

[9] Timpka J, Nitu B, Datieva V, Odin P, Antonini A (2017). Device-Aided Treatment Strategies in Advanced Parkinson’s Disease. International review of neurobiology.

[10] Haney MJ (2015). Exosomes as drug delivery vehicles for Parkinson’s disease therapy. Journal of controlled release: official journal of the Controlled Release Society.

[11] Eyjolfsdottir H (2016). Targeted delivery of nerve growth factor to the cholinergic basal forebrain of Alzheimer’s disease patients: application of a second-generation encapsulated cell biodelivery device. Alzheimer’s research & therapy.

[12] Karami A (2015). Changes in CSF cholinergic biomarkers in response to cell therapy with NGF in patients with Alzheimer’s disease. Alzheimer’s & dementia: the journal of the Alzheimer’s Association.

[13] Ferreira D (2015). Brain changes in Alzheimer’s disease patients with implanted encapsulated cells releasing nerve growth factor. Journal of Alzheimer’s disease: JAD.

[14] Wahlberg LU (2012). Targeted delivery of nerve growth factor via encapsulated cell biodelivery in Alzheimer disease: a technology platform for restorative neurosurgery. Journal of neurosurgery.

[15] Eriksdotter-Jonhagen M (2012). Encapsulated cell biodelivery of nerve growth factor to the Basal forebrain in patients with Alzheimer’s disease. Dementia and geriatric cognitive disorders.

[16] Esquenazi Y (2016). Surgical Resection for Epilepsy Following Cerebral Gunshot Wounds. World neurosurgery.

[17] Crepeau AZ, Sirven JI (2017). Management of Adult Onset Seizures. Mayo Clinic proceedings.

[18] Selden NR (2013). Central nervous system stem cell transplantation for children with neuronal ceroid lipofuscinosis. Journal of neurosurgery. Pediatrics.

[19] Ennezat, P. V. *et al*. From evidence-based medicine to personalized medicine, with particular emphasis on drug-safety monitoring. *Archives of cardiovascular diseases*, 10.1016/j.acvd.2017.01.011 (2017). 10.1016/j.acvd.2017.01.011 28552224

[20] Allen TM, Cullis PR (2004). Drug delivery systems: entering the mainstream. Science.

[21] Mathias NR, Hussain MA (2010). Non-invasive systemic drug delivery: developability considerations for alternate routes of administration. Journal of pharmaceutical sciences.

[22] Kompella UB, Lee VH (2001). Delivery systems for penetration enhancement of peptide and protein drugs: design considerations. Advanced drug delivery reviews.

[23] Langer R (1998). Drug delivery and targeting. Nature.

[24] Alam MI (2010). Strategy for effective brain drug delivery. European journal of pharmaceutical sciences: official journal of the European Federation for Pharmaceutical Sciences.

[25] Pardridge WMCSF (2016). blood-brain barrier, and brain drug delivery. Expert opinion on drug delivery.

[26] Pardridge WM (2005). The blood-brain barrier: bottleneck in brain drug development. NeuroRx: the journal of the American Society for Experimental NeuroTherapeutics.

[27] Neuwelt EA (2011). Engaging neuroscience to advance translational research in brain barrier biology. Nature reviews. Neuroscience.

[28] Yi X, Manickam DS, Brynskikh A, Kabanov AV (2014). Agile delivery of protein therapeutics to CNS. Journal of controlled release: official journal of the Controlled Release Society.

[29] Lockman PR (2003). *In vivo* and *in vitro* assessment of baseline blood-brain barrier parameters in the presence of novel nanoparticles. Pharmaceutical research.

[30] Lockman PR, Mumper RJ, Khan MA, Allen DD (2002). Nanoparticle technology for drug delivery across the blood-brain barrier. Drug development and industrial pharmacy.

[31] Groothuis DR (2000). The blood-brain and blood-tumor barriers: a review of strategies for increasing drug delivery. Neuro-oncology.

[32] Cohen-Pfeffer JL (2017). Intracerebroventricular Delivery as a Safe, Long-Term Route of Drug Administration. Pediatric neurology.

[33] Rossi F (2012). Sustained Delivery of Chondroitinase ABC from Hydrogel System. Journal of functional biomaterials.

[34] Rossi F (2011). Characterization and degradation behavior of agar-carbomer based hydrogels for drug delivery applications: solute effect. International journal of molecular sciences.

[35] Baumann MD (2009). An injectable drug delivery platform for sustained combination therapy. Journal of controlled release: official journal of the Controlled Release Society.

[36] Hoffman AS (2002). Hydrogels for biomedical applications. Advanced drug delivery reviews.

[37] Kanellakopoulou K, Giamarellos-Bourboulis EJ (2000). Carrier systems for the local delivery of antibiotics in bone infections. Drugs.

[38] Killoy WJ (1998). Chemical treatment of periodontitis: local delivery of antimicrobials. International dental journal.

[39] Barry BW (2001). Novel mechanisms and devices to enable successful transdermal drug delivery. European journal of pharmaceutical sciences: official journal of the European Federation for Pharmaceutical Sciences.

[40] Richards Grayson AC (2003). Multi-pulse drug delivery from a resorbable polymeric microchip device. Nature materials.

[41] Zema L, Melocchi A, Maroni A, Gazzaniga A (2017). Three-Dimensional Printing of Medicinal Products and the Challenge of Personalized Therapy. Journal of pharmaceutical sciences.

[42] Hong, N., Yang, G. H., Lee, J. & Kim, G. 3D bioprinting and its *in vivo* applications. *Journal of biomedical materials research. Part B, Applied biomaterials*, 10.1002/jbm.b.33826 (2017). 10.1002/jbm.b.33826 28106947

[43] Ventola CL (2014). Medical Applications for 3D Printing: Current and Projected Uses. P & T: a peer-reviewed journal for formulary management.

[44] Groll J (2016). Biofabrication: reappraising the definition of an evolving field. Biofabrication.

[45] Mironov V, Boland T, Trusk T, Forgacs G, Markwald RR (2003). Organ printing: computer-aided jet-based 3D tissue engineering. Trends in biotechnology.

[46] Biondi M, Ungaro F, Quaglia F, Netti PA (2008). Controlled drug delivery in tissue engineering. Advanced drug delivery reviews.

[47] Zhang YS (2017). 3D Bioprinting for Tissue and Organ Fabrication. Annals of biomedical engineering.

[48] Zhang YS (2016). Bioprinting 3D microfibrous scaffolds for engineering endothelialized myocardium and heart-on-a-chip. Biomaterials.

[49] Agarwala S (2016). A Perspective on 3D BioprintingTechnology: Present and Future. American Journal of Enginering and Applied Sciences.

[50] L. SS (2017). Fabrication of titanium based biphasic scaffold using selective laser melting and collagen immersion. International Journal of Bioprinting.

[51] Lind JU (2017). Instrumented cardiac microphysiological devices via multimaterial three-dimensional printing. Nature materials.

[52] Yi HG (2016). A 3D-printed local drug delivery patch for pancreatic cancer growth suppression. Journal of controlled release: official journal of the Controlled Release Society.

[53] Sun T, Hevner RF (2014). Growth and folding of the mammalian cerebral cortex: from molecules to malformations. Nature reviews. Neuroscience.

[54] Jonathan G, Karim A (2016). 3D printing in pharmaceutics: A new tool for designing customized drug delivery systems. International journal of pharmaceutics.

[55] Bracaglia LG (2017). 3D printing for the design and fabrication of polymer-based gradient scaffolds. Acta biomaterialia.

[56] Torii M, Hashimoto-Torii K, Levitt P, Rakic P (2009). Integration of neuronal clones in the radial cortical columns by EphA and ephrin-A signalling. Nature.

[57] Jones EG, Rakic P (2010). Radial columns in cortical architecture: it is the composition that counts. Cereb Cortex.

[58] GmbH, N. *Nanoscribe Photonic Professional GT Data Sheet*, https://www.nanoscribe.de/files/4514/8179/1302/DataSheet_PPGT_V04_2016_web.pdf (2016).

[59] Kavaldzhiev, M. *et al*. Biocompatbile 3D Printed Magnetic Micro Needles. *Biomed Phys Eng Express* **3**, 10.1088/2057-1976/aa5ccb (2017).

[60] Kim S (2016). Fabrication and Manipulation of Ciliary Microrobots with Non-reciprocal Magnetic Actuation. Scientific reports.

[61] Worthington KS (2017). Two-photon polymerization for production of human iPSC-derived retinal cell grafts. Acta biomaterialia.

[62] Buckmann T (2012). Tailored 3D mechanical metamaterials made by dip-in direct-laser-writing optical lithography. Adv Mater.

[63] Ahmed EM (2015). Hydrogel: Preparation, characterization, and applications: A review. Journal of advanced research.

[64] Nakano M, Nakamura Y, Takikawa K, Kouketsu M, Arita T (1979). Sustained release of sulphamethizole from agar beads. The Journal of pharmacy and pharmacology.

[65] Langer R, Tirrell DA (2004). Designing materials for biology and medicine. Nature.

[66] Langer R (1990). New methods of drug delivery. Science.

[67] Wen Y, Oh JK (2014). Recent strategies to develop polysaccharide-based nanomaterials for biomedical applications. Macromolecular rapid communications.

[68] Oh JK, Drumright R, Siegwart DJ, Matyjaszewski K (2008). The Development of Microgels/Nanogels for Drug Delivery Applications. Progress in Polymer Science.

[69] Wang J (2009). Incorporation of Supramolecular Hydrogels Into Agarose Hydrogels - A Potential Drug Delivery Carrier. Journal of Materials Chemistry.

[70] Laurienzo P (2010). Marine polysaccharides in pharmaceutical applications: an overview. Marine drugs.

[71] d’Ayala GG, Malinconico M, Laurienzo P (2008). Marine derived polysaccharides for biomedical applications: chemical modification approaches. Molecules.

[72] Yoganandan N, Pintar FA (2004). Biomechanics of temporo-parietal skull fracture. Clin Biomech (Bristol, Avon).

[73] Choi HB, Ryu JK, Kim SU, McLarnon JG (2007). Modulation of the purinergic P2X7 receptor attenuates lipopolysaccharide-mediated microglial activation and neuronal damage in inflamed brain. The Journal of neuroscience: the official journal of the Society for Neuroscience.

[74] Blaschke AJ, Weiner JA, Chun J (1998). Programmed cell death is a universal feature of embryonic and postnatal neuroproliferative regions throughout the central nervous system. The Journal of comparative neurology.

[75] Harslof M, Muller FC, Rohrberg J, Rekling JC (2015). Fast neuronal labeling in live tissue using a biocytin conjugated fluorescent probe. Journal of neuroscience methods.

[76] Lanciego JL, Wouterlood FG (2011). A half century of experimental neuroanatomical tracing. Journal of chemical neuroanatomy.

[77] Gewirtz DA, Bristol ML, Yalowich JC (2010). Toxicity issues in cancer drug development. Curr Opin Investig Drugs.

[78] Wicki A, Witzigmann D, Balasubramanian V, Huwyler J (2015). Nanomedicine in cancer therapy: challenges, opportunities, and clinical applications. Journal of controlled release: official journal of the Controlled Release Society.

[79] De Jong WH, Borm PJ (2008). Drug delivery and nanoparticles:applications and hazards. International journal of nanomedicine.

[80] Jernigan TL, Baare WF, Stiles J, Madsen KS (2011). Postnatal brain development: structural imaging of dynamic neurodevelopmental processes. Progress in brain research.

[81] Stiles J, Jernigan TL (2010). The basics of brain development. Neuropsychology review.

[82] Misra A, Ganesh S, Shahiwala A, Shah SP (2003). Drug delivery to the central nervous system: a review. Journal of pharmacy & pharmaceutical sciences: a publication of the Canadian Society for Pharmaceutical Sciences, Societe canadienne des sciences pharmaceutiques.

[83] McGovern RA, Banks GP, McKhann GM (2016). New Techniques and Progress in Epilepsy Surgery. Current neurology and neuroscience reports.

[84] Vasefi F, MacKinnon N, Farkas DL, Kateb B (2017). Review of the potential of optical technologies for cancer diagnosis in neurosurgery: a step toward intraoperative neurophotonics. Neurophotonics.

[85] Hong X (2013). Dissolving and biodegradable microneedle technologies for transdermal sustained delivery of drug and vaccine. Drug design, development and therapy.

[86] Rossi F, Cattaneo E (2002). Opinion: neural stem cell therapy for neurological diseases: dreams and reality. Nature reviews. Neuroscience.

[87] Selden NR, Guillaume DJ, Steiner RD, Huhn SL (2008). Cellular therapy for childhood neurodegenerative disease. Part II: clinical trial design and implementation. Neurosurgical focus.

[88] Guillaume DJ, Huhn SL, Selden NR, Steiner RD (2008). Cellular therapy for childhood neurodegenerative disease. Part I: rationale and preclinical studies. Neurosurgical focus.

[89] Stephens DJ, Allan VJ (2003). Light microscopy techniques for live cell imaging. Science.

[90] Papadopulos F (2007). Common tasks in microscopic and ultrastructural image analysis using Image. J. Ultrastructural pathology.

[91] Son AI, Hashimoto-Torii K, Rakic P, Levitt P, Torii M (2016). EphA4 has distinct functionality from EphA7 in the corticothalamic system during mouse brain development. The Journal of comparative neurology.

